# Evaluating a tobacco-free university policy: A repeated cross-sectional survey of faculty and staff in Lebanon

**DOI:** 10.18332/tid/133751

**Published:** 2021-05-10

**Authors:** Dina Farran, Rima Nakkash, Mahmoud Al-Hindi, Maya Romani, Martin J. O. Asser, Mary Khairallah, Monique Chaaya

**Affiliations:** 1 Department of Health Promotion and Community Health, Faculty of Health Sciences, American University of Beirut, Beirut, Lebanon; 2 Department of Chemical Engineering and Advanced Energy, Maroun Semaan Faculty of Engineering and Architecture, American University of Beirut, Beirut, Lebanon; 3 Department of Family Medicine, American University of Beirut, Beirut, Lebanon; 4 Office of Communications, American University of Beirut, Beirut, Lebanon; 5 Office of Human Resources, American University of Beirut, Beirut, Lebanon; 6 Department of Epidemiology and Population Health, Faculty of Health Sciences, American University of Beirut, Beirut, Lebanon

**Keywords:** tobacco-free policies, smoking behavior, university, faculty, staff

## Abstract

**INTRODUCTION:**

A growing body of research has evaluated the effect of university tobacco-free policies on faculty and staff, however, none of these studies has been carried out in the Eastern Mediterranean Region. This study evaluates changes in faculty and staff attitudes, perceptions and smoking behavior, at 1 year post adoption of a tobacco-free policy in a medium-sized university in Lebanon and the region.

**METHODS:**

Two cross-sectional surveys were conducted in 2017 and 2018: baseline and at 1 year post policy implementation. A random sample of 625 and 624 participants took part in the 2017 and 2018 studies, respectively.

**RESULTS:**

Faculty and staff had a positive attitude towards the policy at the two time points. The belief that there should be exceptions to the policy significantly decreased from 79% to 59% (p=0.002) among all smokers, particularly those with lower educational attainment (81% to 57%, p=0.007). Perception of compliance among peer smokers increased from 73% to 87% (p=0.009). The proportion of smokers did not significantly change at 1 year post policy implementation, however, 44% of smokers with lower educational attainment, compared to only 7% of those with higher educational attainment (p<0.001), reported a decrease in their smoking behavior outside campus.

**CONCLUSIONS:**

The policy had a positive effect on the attitude, behavior and perception of policy benefits among smokers with lower educational attainment, who constitute the majority of smokers. Findings from this study inform and support future efforts to develop university and workplace tobacco-free policies.

## INTRODUCTION

Tobacco, one of the major causes of mortality worldwide, accounts for the death of more than eight million people yearly. Approximately seven million of those deaths are attributed to direct tobacco use while around 1.2 million deaths result from the exposure of non-smokers to secondhand smoke (SHS)^1^. According to the International Labor Organization (ILO), exposure to secondhand smoke at the workplace causes the death of 200000 workers yearly^2^. This is because most working age people spend about a third of their day at work, making interventions in such settings effective in reducing the smoking burden^3^.

In Lebanon, smoking is prohibited in all open public places, public transport, workplaces and outdoor areas of education, health and sports facilities^4^. However, the policy is not properly enforced and the smoking prevalence is still considered among the highest in the Eastern Mediterranean Region. More than 1.3 million adults aged >15 years (37% men and 21% women) were daily smokers in 2015, with the highest proportions aged 40–69 years^5,6^. Data on the exposure of adult non-smokers to secondhand smoke are not available, however, according to the 2011 Global Youth Tobacco Survey (GYTS), 70% of students aged 13–15 years are exposed to secondhand smoke at home and 65% are exposed to it outside home^7^.

Tobacco-free workplaces, including tobacco-free universities, have been effective in decreasing exposure to secondhand smoke, reducing cigarette consumption, increasing smokers’ intention to quit and increasing the likelihood of cessation^8-11^. Non-smokers’ exposure to secondhand smoke decreased by 28% in workplaces banning tobacco smoking in the US^12^. These policies are also associated with a 4% decrease in the total prevalence of tobacco consumption and 29% reduction in the total consumption per employee according to a systematic review including smoke-free workplaces in the United States, Australia, Canada and Germany^8^. Chapman et al.^13^ found that the number of daily smoked cigarettes decreased by 3.5 cigarettes per smoker in workplaces having these restrictions in Australia and the US^13^. Such workplaces not only provide a supportive environment for smokers willing to quit but also decrease the likelihood of initiation^14^. They result in positive changes in the social norms associated with smoking that can disseminate to other environments such as employees’ homes^15^. The reduced smoking behavior results from the inconvenience of leaving the work area to smoke, from the negative image of behaviors attributed to nicotine dependence or from the reduction of smoking cues (as other colleagues are not witnessed smoking)^14^. In addition to their effect on the smoking behavior, these policies increase productivity and decrease absenteeism rates as smokers miss more work days due to illness^16,17^. Salti et al.^17^ reported that ex-smokers and smokers’ absenteeism in Lebanon leads to a loss of production of $102.2 million per year.

Workers have generally shown support for smoking bans at the workplace, although non-smokers typically have a more favorable attitude towards them^8,11^. In college and university settings, compliance to tobacco-free policies among staff and faculty members has been substantial and support has ranged between 64% and 76%^18^. However, the effect of tobacco-free policies has been much dependent on their strength, meaning that comprehensive tobacco policies decrease smoking prevalence and cigarette consumption twice as much as partial bans^8^. Demographic factors, such as workers’ socioeconomic status (SES), represented by education level, and occupation and income, were also found associated with the effectiveness of the policy in reducing the smoking behavior as workers with higher SES have been reported to have higher quit attempts and cessation rates^19-21^. Additionally, Mamudu et al.^22^ reported that faculty members with higher educational attainment and income are more likely to support university tobacco control policies. Thus, investigating the changes in the attitude and smoking behavior of employees with lower educational attainment particularly, could be key in determining the effectiveness of the tobacco control measures.

The American University of Beirut (AUB) is one of the largest employers in Lebanon with more than 5635 staff and faculty members. The university started its tobacco-free policy initiative in 2008 by banning smoking in all university buildings except faculty apartments. In January 2018, AUB adopted a comprehensive tobacco-free policy as instituted by national policy, although it was the first Lebanese higher educational institution to do so. AUB tobacco-free policy prohibits: smoking any kind of tobacco (including cigarettes, cigars, pipe tobacco, e-cigarettes or any other electronic smoking devices, waterpipes, hookah tobacco, snuff, chewing tobacco, dipping tobacco, bidis, blunts, and clove cigarettes) in all indoor and outdoor areas on campus and in university operated facilities off-campus, selling or promoting tobacco and tobacco-related products on university premises, and accepting research funds or event sponsorships from tobacco or tobacco-related companies. Signage, banners and large promotional cubes with messages about the tobacco-free policy were placed at all university gates, major buildings, halls, gathering places and on all AUB social media platforms and screens. Recycling receptacles were placed at all entry points to the university, which later on developed into a cigarette litter-recycling program. The recycled cigarette filters were used to form paddleboards. Free access to a smoking cessation program, including counseling sessions and nicotine replacement therapy, was also made available for students, staff or faculty wishing to quit^23^.

Here, we report specifically on the attitudes, perceptions and behavior changes amongst university faculty and staff at 1 year post adoption of the tobacco-free policy. This is done by examining more closely differences in attitudes, perceptions and behaviors between smokers and non-smokers as well as by educational attainment of faculty and staff surveyed. To our knowledge, this study is the first to evaluate the effectiveness of a university tobacco-free policy on employees in the context of Lebanon and the region. It was hypothesized that there would be a higher level of support, an improvement in the perception of policy benefits and a positive change in the smoking behavior.

## METHODS

### Study design

This study is based on two repeated cross-sectional surveys. The first was conducted prior to the implementation of the smoke-free policy (December 2017) while the second was done one year after (December 2018). A sample of 1722 staff (non-academic employees who perform administrative or support work) and 960 faculty members (academics who perform educational functions) was randomly selected in each round. Faculty and staff were sent invitations by emails and were asked to access a survey link if they considered participating. To ensure participation of lower grade staff (who rarely use their email accounts), hard copies of the questionnaire were also placed for two weeks in the administration offices of almost all departments. Completed surveys were deposited in a locked box. Where needed, a research assistant from the Faculty of Health Sciences was available to assist staff, wishing to participate, in filling in the questionnaire. The overall faculty and staff response rate was about 23% in both years (625 participated in 2017, and 624 in 2018). The number of staff who completed the hard copy version of the survey was 129 in 2017, and 169 in 2018. No assistance was provided to participants in the first cross-sectional study, however, in the second one a research assistant helped 71 participants (mainly grades 1 and 2) in filling in the questionnaire. The Institutional Review Board at AUB approved the research protocol. Participation was voluntary and data were kept anonymous.

### Measures

The 55-question survey took 5–7 minutes to complete. The questionnaire asked about demographic information, faculty and staff attitude towards the smoking policy, perceived benefits, and smoking behavior. To ensure appropriateness and clarity of the survey, pre-testing was done on a small number (n=20) of staff and faculty members before invitations were sent. No modifications were made to the questionnaire since the feedback was all positive.

### Demographics

Information on gender, age, marital status, and number of children, was collected. Participants were asked to identify their primary role (staff or faculty) and their educational attainment [primary (grades 1–6), intermediate (grades 7–9), technical, secondary (grades 10–12), Bachelor’s, Master’s, and doctoral degrees].

### Smoking behavior

Information on the smoking status, history, and frequency, was collected. The smoking status, initially divided into four categories (non-smokers, ex-smokers, occasional smokers, and regular smokers) was grouped into two: smokers (current) and non-smokers (never smoked, former, and occasional smoker). Participants, identifying themselves as smokers, were further asked if they considered themselves addicted, had concerns about the health effects of smoking, had intentions to quit, had made quit attempts, and considered participating in a cessation program. In addition, smokers had to report any changes (increase, decrease, remained the same) in smoking intensity post policy implementation. Items related to the smoking behavior in the survey were adapted from the International Tobacco Control (ITC) questionnaire^24^.

### Attitude towards the smoking policy

Participants’ attitude towards the policy was assessed by the extent of support, and the extent to which they believed that the university tobacco-free policy had created a healthy environment, and promoted quit attempts. In addition, they were asked whether there should be exceptions to the policy. Items related to attitude in the survey were created uniquely for this project. All responses were initially reported on a 4-point Likert scale (large extent, some extent, not at all, not sure) then dichotomized into Yes or No (large extent and some extent were considered Yes, while not at all and not sure were considered as No).

### Perception of compliance and benefits

In this section, participants had to determine whether they perceived their peers as being compliant or not compliant with the policy. They were also asked to determine the extent to which they perceived the following as policy benefits: reduction in smoking frequency, increase in faculty and staff productivity, decrease in rate of faculty and staff sick days, and decrease in rate of student absences. Items related to the perception of compliance and benefits in the survey were created uniquely for this project. Responses were first reported on a 5-point Likert scale (not a benefit, minor benefit, moderate benefit, major benefit, and don’t know) then dichotomized into Yes or No (minor benefit, moderate benefit, and major benefit, were considered Yes, while not a benefit and don’t know were considered as No).

### Statistical analysis

Based on their educational attainment, smokers were stratified into participants with lower educational attainment (<Bachelor’s degree, BD) and higher educational attainment (≥BD). The χ^2^ test was used to determine differences in attitude, perceived compliance, and perceived policy benefits at baseline and 1 year post policy implementation among smokers and non-smokers. The same analysis was repeated on smokers stratified by educational attainment. Significant differences were identified at a p<0.05. All the statistical analyses were performed using the statistical software R version 3.4.1.

## RESULTS

Of the faculty and staff who were invited to participate, a larger proportion of females completed the questionnaire in the first cross-sectional survey compared to the follow-up survey (56% compared to 49%). The distribution of participants by faculty and staff was similar in both years. The smoking status did not significantly vary with 18% and 21% of participants being smokers in 2017 and 2018, respectively (Table 1). When stratified by educational attainment, the proportion of smokers with higher educational attainment decreased from 41% to 35% at 1 year post policy implementation, yet the change was not statistically significant (Table 1).

**Table 1:** Characteristics of faculty and staff at baseline and at 1 year post policy implementation

*Characteristics*	*Stage of survey implementation*		p*
	*Baseline, 2017 (n=625) n (%)*	*At 1 year post, 2018 (n=624) n (%)*	
**Gender** (Female)	348 (56.1)	299 (48.9)	0.014
**Age** (years), mean (SD)	41.21 (11.53)	39.61 (11.50)	0.021
**Type of respondent**			0.183
Faculty	211 (33.8)	184 (30.1)	
Staff	414 (66.2)	428 (69.9)	
**Staff education level**			<0.001
Primary	26 ( 6.5)	46 (10.9)	
Intermediate	54 (13.4)	72 (17.1)	
Technical	26 ( 6.5)	36 ( 8.5)	
Secondary	31 ( 7.7)	64 (15.2)	
Bachelor’s	121 (30.1)	98 (23.2)	
Master’s	141 (35.1)	98 (23.2)	
Doctoral	3 ( 0.7)	8 ( 1.9)	
**Smoking status**			0.348
Non-smoker	496 (81.6)	486 (79.3)	
Smoker	112 (18.4)	127 (20.7)	
**Faculty and staff who are smokers**			0.417
With lower educational attainment	65 (59.1)	82 (65.1)	
With higher educational attainment	45 (40.9)	44 (34.9)	

Number applicable for ‘faculty and staff who are smokers’: 112 for 2017, and 127 for 2018. Totals do not add up because of missing values.

* p<0.05 considered significant.

### Attitude towards the policy

Overall, participants had a positive attitude towards the tobacco-free policy with little changes between 2017 and 2018, and with a consistently and significantly higher proportion of non-smokers supporting it. The proportion of smokers supporting the policy and believing that it had created a healthy environment positively changed over time, with differences not being statistically significant. However, the proportion of smokers believing that there should be exceptions to the policy significantly decreased (from 79% in 2017 to 59% in 2018, p=0.002), particularly among those with lower educational attainment (from 80% to 64%, p=0.007) (Table 2).

**Table 2:** Attitude at baseline and at 1 year post policy implementation by smoking status and educational attainment

*Attitude*	*All participants*			*Participants who are smokers*		
	*Non-smokers (NS) n (%)*	*Smokers (S) n (%)*	*P (NS/S)*	*With lower educational attainment (<BD) n (%)*	*With higher educational attainment (≥BD) n (%)*	p* (<BD)/(≥BD)
**Support the policy**						
Baseline (B)	452 (91.5)	70 (66.0)	<0.001	49 (81.7)	19 (43.2)	<0.001
1 year post (1YP)	459 (94.6)	93 (73.2)	<0.001	65 (79.3)	27 (61.4)	0.051
p (B/1YP)	0.071	0.294		0.888	0.135	
**Think the policy has created a healthy environment**						
Baseline	444 (90.1)	70 (66.0)	<0.001	45 (76.3)	24 (53.3)	0.025
1 year post	438 (90.3)	96 (75.6)	<0.001	64 (78.0)	31 (70.5)	0.467
p (B/1YP)	0.982	0.145		0.964	0.149	
**Think the policy has promoted quit attempts**						
Baseline	352 (72.1)	56 (52.3)	<0.001	42 (70.0)	13 (28.9)	<0.001
1 year post	289 (59.5)	64 (50.4)	0.082	52 (63.4)	11 (25.0)	<0.001
p (B/1YP)	<0.001	0.869		0.522	0.862	
**Think there should be exceptions to the policy**						
Baseline	220 (44.6)	82 (78.8)	<0.001	46 (80.7)	36 (80.0)	0.999
1 year post	100 (20.6)	75 (59.1)	<0.001	47 (57.3)	28 (63.6)	0.618
p (B/1YP)	<0.001	0.002		0.007	0.138	

Percentages may not reflect the figures as there were some missing values.

* p<0.05 considered significant.

### Perception of compliance and benefits

Perception of policy benefits were reported more by non-smokers compared with smokers. In 2018, 78% of non-smoker participants believed that the policy had contributed to a reduction in the smoking frequency compared to 59% of smokers (p<0.001). Similarly, more non-smokers in 2018 thought that the policy had led to an increase in faculty and staff productivity (60% vs 43%, p=0.001) and a decrease in their sickness (51% vs 36%, p=0.003) compared to smokers. Stratified by educational attainment, the perception of policy benefits was reported more by smokers with lower educational attainment. In 2018, 51% of smokers with less than a Bachelor’s degree compared to 27% of smokers with more than a Bachelor’s degree (p=0.016) thought that the policy increased their productivity, and 48% compared to 16% (p=0.001) thought that the policy decreased their sickness. However, no significant changes were seen between 2017 and 2018 in both categories.

As for compliance, the proportion of smokers perceiving students and staff as being compliant with the policy significantly increased between 2017 and 2018 (61% to 76% for students, p=0.021; and 73% to 87% for staff, p=0.009). This change was particularly noticed among smokers with lower educational attainment (Table 3).

**Table 3:** Policy perceived benefits at baseline and at 1 year post implementation by smoking status and educational attainment

*Perceived benefits*	*All participants*			*Participants who are smokers*		
	*Non-smokers (NS) n (%)*	*Smokers (S) n (%)*	*p (NS/S)*	*With lower educational attainment (<BD) n (%)*	*With higher educational attainment (≥BD) n (%)*	p* (<BD)/(≥BD)
**Think the policy reduced smoking frequency**						
Baseline (B)	426 (86.6)	74 (69.8)	<0.001	38 (63.3)	35 (77.8)	0.168
1 year post (1YP)	375 (77.5)	75 (59.1)	<0.001	49 (59.8)	26 (59.1)	0.999
p (B/1YP)	<0.001	0.117		0.797	0.095	
**Think the policy decreased students’ absences**						
Baseline	281 (57.5)	34 (33.3)	<0.001	24 (43.6)	10 (22.2)	0.042
1 year post	207 (42.7)	38 (29.9)	0.012	32 (39.0)	6 (13.6)	0.006
p (B/1YP)	<0.001	0.682		0.718	0.436	
**Think the policy increased faculty and staff productivity**						
Baseline	306 (62.8)	48 (46.2)	0.002	28 (49.1)	20 (44.4)	0.787
1 year post	292 (60.2)	54 (42.5)	0.001	42 (51.2)	12 (27.3)	0.016
p (B/1YP)	0.438	0.674		0.944	0.142	
**Think the policy decreased faculty and staff sick days**						
Baseline	327 (66.6)	41 (39.4)	<0.001	30 (52.6)	11 (24.4)	0.007
1 year post	249 (51.3)	46 (36.2)	0.003	39 (47.6)	7 (15.9)	0.001
p (B/1YP)	<0.001	0.716		0.678	0.46	
**Think students are compliant**						
Baseline	391 (79.6)	65 (61.3)	<0.001	34 (55.7)	30 (68.2)	0.277
1 year post	372 (76.7)	96 (76.2)	0.998	66 (81.5)	30 (68.2)	0.144
p (B/1YP)	0.302	0.021		0.002	0.999	
**Think staff are compliant**						
Baseline	414 (84.8)	79 (73.1)	0.006	41 (66.1)	37 (82.2)	0.103
1 year post	398 (82.1)	111 (87.4)	0.194	71 (86.6)	40 (90.9)	0.67
p (B/1YP)	0.281	0.009		0.007	0.374	
**Think faculty are compliant**						
Baseline	407 (82.6)	75 (70.8)	0.008	40 (66.7)	35 (77.8)	0.304
1 year post	360 (74.5)	96 (75.6)	0.897	65 (79.3)	31 (70.5)	0.375
p (B/1YP)	0.003	0.495		0.135	0.584	

Percentages may not precisely reflect the figures as there were some missing values.

* p<0.05 considered significant.

### Smoking behavior change

Although the proportion of regular smokers was similar in both cross-sectional studies, the proportion of those with lower educational attainment was higher in both years (59% vs 41% in 2017; and 65% vs 35% in 2018). A decrease in the smoking behavior, though not statistically significant, was only noted among smokers with higher educational attainment. However, 44% of smokers with lower educational attainment reported a decrease in their off-campus smoking behavior compared to only 7% of those with higher educational attainment (p<0.001). A small proportion of the smoking participants joined the smoking cessation program at AUB and a high proportion (50% of those with lower and 36% of those with higher educational attainment) considered participating in such programs (Table 4).

**Table 4:** Smoking behavior at baseline and at 1 year post policy implementation by educational attainment

*Smoking behavior*	*Participants who are smokers*		
	*With lower educational attainment (<BD) n (%)*	*With higher educational attainment (≥BD) n (%)*	p*
**Thinking of quitting within the next 6 months**			
Baseline (B)	42 (65.6)	18 (40.0)	0.014
1 year post (1YP)	49 (59.8)	18 (40.9)	0.067
p (B/1YP)	0.58	0.999	
**Consider participating in a smoking cessation program**			
Baseline	36 (57.1)	21 (47.7)	0.445
1 year post	41 (50.0)	16 (36.4)	0.201
p (B/1YP)	0.492	0.388	
**Joined the smoking cessation program**			
Baseline	NA	NA	
1 year post	5 (6.1)	2 (4.5)	0.663
p (B/1YP)	-	-	
**Decreased off-campus smoking behavior**			
Baseline	NA	NA	
1 year post	36 (43.9)	3 (6.8)	<0.001
p (B/1YP)	-	-	

Percentages may not precisely reflect the figures as there were some missing values.

* p<0.05 considered significant. NA: not applicable.

## DISCUSSION

This study reports on the change of attitude, perceived benefits and smoking behavior of staff and faculty at 1 year post implementation of a tobacco-free policy in a university in the Middle East. We found a significant effect of the policy on smokers, particularly those with lower educational attainment. Although the smoking prevalence did not significantly change, the policy had an effect on the smoking behavior as a large proportion of participants reported a decrease in their smoking frequency outside campus, supporting the hypothesis made at the beginning of the study.

Consistent with many US studies, the majority of faculty and staff in both surveys had a positive attitude towards the policy with non-smokers always showing greater support and the proportion of smokers favoring such policy on campus significantly increasing post policy implementation^18,22,25-27^. Other studies, however, reported that smokers and ex-smokers are more likely to oppose and violate tobacco control policies^28-30^. Although ex-smokers could be more supportive, they still believed in the freedom of choice and showed sympathy towards current smokers’ need for tobacco^28-30^. The proportion of smokers with lower educational attainment believing that there should be exceptions to the policy significantly decreased at 1 year post policy implementation, suggesting an improvement in the attitude of smokers known to be less likely to support such policies. The improvement seen in this particular group of employees suggests that educational institutions could be considered as important venues to develop strong and effective tobacco-control policies even in environments generally known for being tobacco friendly. As for the perception of compliance, results were in line with previous studies showing that when the policy is properly enforced, compliance increases over time^31^. The change in social norms resulting from the implementation of the tobacco-free policy could lead to a shift towards a more socially accepted behavior, at least on campus^32^. The fact that the overall smoking prevalence did not significantly change at 1 year post policy implementation may be attributed to the pro-tobacco environment and the weak enforcement of the tobacco control law at the national level. However, the high proportion of smokers reporting a decrease in their smoking frequency outside campus suggests some effect of the policy on smoking behavior. These results are promising, as Lechner et al.^9^ reported, in their study assessing changes in smoking prevalence over four years post implementation of a university tobacco-free policy, that the decrease was not significant until the second year, thus, illustrating the importance of examining changes over a longer period.

Although great effort has been made to understand the attitude and perception of students towards university tobacco control policies, little is known about the attitude of faculty and staff, the most stable population, towards such policies, especially in the Eastern Mediterranean Region. Few studies have investigated the general attitude of faculty and staff towards such policies in the region, however, none has looked for a change post policy implementation or has tested for policy effectiveness^33,34^. This study could set the stage for similar research in the region, thus informing future development of legislation and reducing overall tobacco consumption.

### Strengths and limitations

This study has some strengths as well as limitations. First, the large sample size in both cross-sectional surveys gives power to the tests in detecting variations in attitude, perceived benefits and smoking behavior at 1 year post policy implementation. Second, this study adds to the literature gap in evaluations of university tobacco-free policies in countries outside the US and in low- and middle-income countries. Third, knowing the tobacco-friendly environment in the Middle East, findings of this study may be generalized to other private universities in Lebanon and the region. As for the limitations, the cross-sectional nature makes it hard to infer any causal association between the variables, and the repeated design cannot detect individual changes of attitudes and behavior. However, many studies in the literature and worldwide have used the repeated cross-sectional design to measure the effect of tobacco policy or introduction of new tobacco products on attitudes toward tobacco and tobacco consumption^33,35^. Selection bias is another potential limitation of the study, however, the random selection of the participants in a similar manner from the same underlying populations minimizes this bias. The fact that there was oversampling of females in the first survey due to the higher response rate among them is unlikely to affect the results as we did not anticipate any effect of changes on behavior or attitude.

## CONCLUSIONS

The university tobacco-free policy had a positive effect on the attitude, behavior and perception of policy benefits among smokers with lower educational attainment, who constitute the majority of smokers. This study highlights the effectiveness of tobacco-control policies in universities and promotes this setting as an important venue for the implementation of such policies. In the Eastern Mediterranean Region, known for being generally tobacco-friendly, all educational institutions should consider implementing such university tobacco-free policies to support national tobacco control regulatory measures in place.
